# Supplementary material for: RB loss sensitizes cells to replication-associated DNA damage after PARP inhibition by trapping
Source: Life Sci Alliance. 2023 Sep 13;6(12):e202302067. doi: 10.26508/lsa.202302067 (PMC10500056; doi:10.26508/lsa.202302067)
Supplement: Supplementary file 1 [file LSA-2023-02067_TableS1.docx]

**Supplemental Table S1. DNA damage parameters of screened epigenetic modulators**

| Target | Drug | DNA damage  (Fold-change relative  to untreated control) | | Robust  Z-score |
| --- | --- | --- | --- | --- |
|  |  | Control | shRB |  |
| AURK | CYC116 | 0.11 | 1.64 | 20.04 |
| AURK | JNJ-7706621 | 2.01 | 4.52 | 3.12 |
| AURK | PHA-680632 | 1.31 | 2.70 | 2.87 |
| AURK | VX-680 (Tozasertib) | 3.39 | 3.83 | 1.56 |
| AURK | MK-5108 (VX-689) | 1.52 | 1.69 | 1.54 |
| AURK | CCT129202 | 2.00 | 2.22 | 1.53 |
| AURK | Danusertib (PHA-739358) | 0.96 | 0.96 | 1.38 |
| AURK | MLN8054 | 1.82 | 1.78 | 1.35 |
| AURK | Barasertib (AZD1152-HQPA) | 2.51 | 2.14 | 1.18 |
| AURK | Aurora A Inhibitor I | 2.45 | 1.80 | 1.02 |
| AURK | ZM 447439 | 1.87 | 1.35 | 1.00 |
| AURK | Alisertib (MLN8237) | 2.20 | 1.40 | 0.88 |
| AURK | SNS-314 Mesylate | 2.29 | 1.21 | 0.73 |
| AURK | AMG-900 | 3.69 | 1.85 | 0.70 |
| BRD4 | PFI-1 (PF-6405761) | 1.47 | 4.81 | 4.52 |
| BRD4 | Bromosporine | 0.85 | 2.02 | 3.29 |
| BRD4 | RVX-208 | 0.68 | 1.15 | 2.35 |
| BRD4 | CPI-203 | 0.74 | 1.08 | 2.02 |
| BRD4 | UNC1215 | 0.36 | 0.45 | 1.72 |
| BRD4 | I-BET151 (GSK1210151A) | 1.67 | 1.69 | 1.40 |
| BRD4 | I-BET-762 | 1.33 | 1.30 | 1.35 |
| BRD4 | SGC-CBP30 | 0.69 | 0.64 | 1.28 |
| BRD4 | OTX015 | 2.94 | 1.65 | 0.78 |
| BRD4 | (+)-JQ1 | 1.87 | 0.84 | 0.62 |
| DNMT | Zebularine | 0.57 | 2.25 | 5.45 |
| DNMT | Azacitidine | 0.19 | 0.70 | 5.24 |
| DNMT | SGI-1027 | 0.43 | 1.36 | 4.36 |
| DNMT | RG108 | 0.49 | 0.68 | 1.94 |
| DNMT | Lomeguatrib | 1.30 | 0.80 | 0.86 |
| EZH2 | 3-Deazaneplanocin A (DZNeP) | 0.36 | 1.78 | 6.88 |
| EZH2 | EPZ-6438 (Tazemetostat) | 0.34 | 1.15 | 4.66 |
| EZH2 | EPZ5676 | 0.48 | 1.18 | 3.39 |
| EZH2 | Entacapone | 0.84 | 1.33 | 2.20 |
| EZH2 | MM-102 | 0.67 | 1.01 | 2.07 |
| EZH2 | EPZ004777 | 0.67 | 0.76 | 1.56 |
| EZH2 | SGC 0946 | 0.47 | 0.50 | 1.49 |
| HDAC | RGFP966 | 0.92 | 2.89 | 4.36 |
| HDAC | Rocilinostat (ACY-1215) | 1.35 | 3.58 | 3.66 |
| HDAC | Resminostat | 1.06 | 2.71 | 3.53 |
| HDAC | Givinostat (ITF2357) | 3.75 | 5.36 | 1.98 |
| HDAC | Scriptaid | 1.97 | 2.19 | 1.54 |
| HDAC | Belinostat (PXD101) | 5.12 | 5.08 | 1.37 |
| HDAC | PCI-24781 (Abexinostat) | 1.76 | 1.66 | 1.31 |
| HDAC | Trichostatin A (TSA) | 7.47 | 6.17 | 1.14 |
| HDAC | Panobinostat (LBH589) | 10.72 | 6.17 | 0.80 |
| HDAC | TMP269 | 1.08 | 0.59 | 0.75 |
| HDAC | Entinostat (MS-275) | 5.53 | 2.64 | 0.66 |
| HDM | GSK J4 HCl | 1.65 | 6.44 | 5.40 |
| HDM | IOX1 | 0.53 | 1.09 | 2.86 |
| HDM | Tranylcypromine (2-PCPA) HCl | 1.05 | 1.29 | 1.70 |
| HDM | OG-L002 | 0.91 | 1.02 | 1.56 |
| JAK | AZD1480 | 0.51 | 2.52 | 6.91 |
| JAK | Ruxolitinib (INCB018424) | 0.34 | 1.09 | 4.42 |
| JAK | Gandotinib (LY2784544) | 2.49 | 5.67 | 3.15 |
| JAK | Tofacitinib (CP-690550) | 1.19 | 1.93 | 2.24 |
| JAK | CEP-33779 | 0.46 | 0.31 | 0.92 |
| JAK | CYT387 | 2.92 | 1.33 | 0.63 |
| PARP1/2 | Olaparib (AZD2281) | 1.14 | 3.10 | 3.76 |
| PARP1/2 | Talazoparib (BMN 673) | 4.64 | 11.24 | 3.35 |
| PARP | Rucaparib (AG-014699) | 1.39 | 2.61 | 2.60 |
| PARP1 | AG-14361 | 0.97 | 1.71 | 2.44 |
| PARP | PJ34 HCl | 0.45 | 0.70 | 2.14 |
| PARP1/2 | Veliparib (ABT-888) | 1.74 | 2.43 | 1.93 |
| PARP | 3-Aminobenzamide | 0.92 | 0.88 | 1.32 |
| PARP | AZD2461 | 0.72 | 0.64 | 1.24 |
| PARP | INO-1001 | 0.47 | 0.36 | 1.07 |
| Other | SMI-4a | 0.69 | 2.09 | 4.21 |
| Other | C646 | 0.36 | 0.64 | 2.49 |
| Other | Quercetin | 1.34 | 2.36 | 2.44 |
| Other | AZD1208 | 0.46 | 0.80 | 2.41 |
| Other | Resveratrol | 0.90 | 0.99 | 1.53 |
| Other | Sirtinol | 0.98 | 1.07 | 1.50 |
| Other | FG-4592 | 1.02 | 1.01 | 1.37 |
| Other | EX 527 (Selisistat) | 1.08 | 1.03 | 1.32 |
| Other | Iniparib (BSI-201) | 1.58 | 1.26 | 1.10 |
| Other | Procainamide HCl | 0.96 | 0.65 | 0.94 |
| Other | IOX2 | 0.86 | 0.56 | 0.90 |
| Other | CX-6258 HCl | 7.10 | 0.00 | 0.00 |
