# Supplementary material for: RB loss sensitizes cells to replication-associated DNA damage after PARP inhibition by trapping
Source: Life Sci Alliance. 2023 Sep 13;6(12):e202302067. doi: 10.26508/lsa.202302067 (PMC10500056; doi:10.26508/lsa.202302067)
Supplement: Supplementary file 2 [file LSA-2023-02067_TableS2.docx]

**Supplemental Table S2. Survival IC_50_ values for screen hits and additional PARP inhibitors**

| Target | Drug | Survival IC_50_ (μM) | | IC_50_ ratio  (Control/shRB) |
| --- | --- | --- | --- | --- |
|  |  | Control | shRB |  |
| AURK | CYC116 | 2.57 | 1.78 | 1.44 |
| AURK | JNJ-7706621 | 1.36 | 1.28 | 1.06 |
| BRD4 | PFI-1 (PF-6405761) | 13.52 | 9.89 | 1.37 |
| BRD4 | Bromosporine | 4.33 | 2.65 | 1.63 |
| EZH2 | DZNeP | >100 | >100 | NA |
| EZH2 | Tazemetostat | 0.34 | 0.75 | 0.45 |
| HDAC | RGFP966 | 56.62 | 29.49 | 1.92 |
| HDAC | Rocilinostat | 8.26 | 4.63 | 1.78 |
| HDAC | Resminostat | 4.80 | 2.35 | 2.04 |
| JAK | AZD1480 | 40.88 | 20.75 | 1.97 |
| JAK | Ruxolitinib | 5.52 | 3.25 | 1.70 |
| JAK | Gandotinib | ≳100 | 10.78 | NA |
| PARP1/2 | Olaparib | 4.78 | 1.74 | 2.74 |
| PARP1/2 | Talazoparib | 0.28 | 0.02 | 14.00 |
| PARP | Rucaparib | 3.28 | 0.80 | 4.10 |
| PARP1 | AG-14361 | 24.46 | 5.64 | 4.33 |
| PARP1/2 | Veliparib | 57.24 | 13.07 | 4.38 |
| PARP | 3-Aminobenzamide | >100 | >100 | NA |
| PARP | AZD2461 | 22.15 | 6.00 | 3.69 |
| PARP | INO-1001 | >100 | >100 | NA |
| PARP | PJ34-HCl | 71.41 | 42.21 | 1.69 |

(NA) Not applicable
