# Supplementary material for: RB loss sensitizes cells to replication-associated DNA damage after PARP inhibition by trapping
Source: Life Sci Alliance. 2023 Sep 13;6(12):e202302067. doi: 10.26508/lsa.202302067 (PMC10500056; doi:10.26508/lsa.202302067)
Supplement: Supplementary file 3 [file LSA-2023-02067_TableS3.docx]

**Supplemental Table S3. Drugs and antibodies used**

| Reagent | Company | Catalog number | Concentration |
| --- | --- | --- | --- |
|  |  |  |  |
| Doxycycline | Thermo Fisher | 446061000 | 2µg/mL |
| Epigenetic Modulator Library | Selleck Chemicals | Z113676 | 10µM |
| Olaparib | Selleck Chemicals | S1060 | 2.5µM |
| Rucaparib | Selleck Chemicals | S1098 | 2.5µM |
| Talazoparib | Selleck Chemicals | S7048 | 20nM |
| Veliparib | Selleck Chemicals | S1004 | 10µM |
| PARG inhibitor | Tocris | 5952 | 10µM |
| Emetine | Sigma | E2375 | 2µM |
| Acridine Orange | Thermo Fisher | A-3568 | 100µg/mL |
| Mouse-RB (clone 4H1) | Cell Signaling | 9009L | 1:1000 dil |
| Mouse-GAPDH | Proteintech | 600004-1 | 1:100000 dil |
| Mouse- α-tubulin | Santa Cruz | sc-32293 | 1:1000 dil |
| HRP-labeled anti-mouse | Cytiva | NA931V | 1:5000 dil |
| Rabbit-γH2AX | Cell Signaling | 2577L | 1:1000 dil |
| Mouse-γH2AX | Millipore | 05-636 | 1:500 dil |
| Rabbit-pRPA | Sigma | PLA0310 | 1:500 dil |
| Human-ACA | Antibodies Inc. | 15-234 | 1:500 dil |
| Mouse-PAR | Trevigen | 4335-MC-100 | 1:250 dil |
| Alexa Fluor 488 anti-rabbit | Thermo Fisher | A-11008 | 1:1000 dil |
| Alexa Fluor 488 anti-human | Thermo Fisher | A-11013 | 1:1000 dil |
| Alexa Fluor 546 anti-rabbit | Thermo Fisher | A-10040 | 1:1000 dil |
| Alexa Fluor 546 anti-mouse | Thermo Fisher | A-11003 | 1:1000 dil |
| Alexa Fluor 680 anti-rabbit | Thermo Fisher | A-21076 | 1:1000 dil |
| Alexa Fluor 680 anti-mouse | Thermo Fisher | A-21058 | 1:1000 dil |
